# Structural Optimization of Graphene Triangular Lattice Phononic Crystal Based on Dissipation Dilution Theory

**DOI:** 10.3390/nano12162807

**Published:** 2022-08-16

**Authors:** Xiande Zheng, Ying Liu, Jing Qiu, Guanjun Liu

**Affiliations:** College of Intelligence Science and Technology, National University of Defense Technology, Changsha 400713, China

**Keywords:** graphene, phononic crystal, dissipation dilution, finite simulation

## Abstract

Nanomechanical resonators offer brilliant mass and force sensitivity applied in many fields, owing to a low mass m and high-quality factor Q. However, in vibrating process, resonant energy is inevitably dissipated. Typically, quality factor does not surpass the inverse of the material loss angle φ. Recently, some exceptions emerged in the use of highly stressed silicon nitride material. As yet, it is interpreted that the pre-stress seems to “dilute” the intrinsic energy dissipation according to the Zener model. Is there any other material that could further break the 1/φ limit and achieve higher quality factors? In our previous research, through theoretical calculation and finite element simulation, we have proved that graphene’s quality factor is two orders of magnitude larger than silicon nitride, on account of the extremely thin thickness of graphene. Based on this, we further optimize the structure of phononic crystals to achieve higher quality factors, in terms of duty cycle and cell size. Through simulation analysis, the quality factor could improve with a larger duty cycle and bigger cell size of triangular lattice phononic crystal. Unexpectedly, the Q amplification coefficient of the 3 × 5-cell structure, which is the least number to compose a phononic crystal with a central defect area, is the highest. In contrast, the minimal cell-number structure in hexagonal lattice could not achieve the brilliant dissipation dilution effect as well as the triangular one. Then we consider how overall size and stress influence quality factor and, furthermore, compare theoretical calculation and finite simulation. Lastly, we start from the primitive 3 × 5 cells, constantly adding cells to the periphery. Through simulation, to our surprise, the largest Q amplification coefficient does not belong to the largest structure, instead originating from the moderate one consisting of 7 × 13 cells.

## 1. Introduction

In the field of mass and force measurement, nanomechanical resonators could offer brilliant sensitivity, applied in quantum [1] and classical [2] signal measurements, magnetic single spin [3] and single-protein mass sensing [4], gravitational waves detectors [5] and many other sensing applications [6,7,8]. A low mass m and high quality factor Q endow nanomechanical resonators with these superior characteristics. Considering even very small external disturbances, low mass could bring about relatively large resonant frequency changes; simultaneously, high quality factor guarantees that random fluctuations can be squeezed to a very small extent in the vibrating process. Nevertheless, virtually, quality factor conventionally does not exceed the inverse of the material loss angle φ, strongly associated with resonant losses [9], otherwise known as energy dissipation.

According to the dissipation theorem [10], dissipation gives rise to noise, imposing constraints on the resonant sensitivity and frequency stability. How to decrease the resonant dissipation is a very complicated issue, on account of not-well-understood intrinsic and external loss mechanisms. The concept of dissipation dilution was put forward by Gonzalez et al. [11,12], who ascribed the elastic energy loss of suspension wire to the diluted effect from the conservative gravitational potential. Afterwards, highly strained nano strings and membranes without external potential displayed analogous [13,14,15]. In subsequent studies, the quality factor physical models of uniform beams [16] and membranes [17] have been set up; through the comprehensive calculation of structural mechanics, the Q of highly strained resonator was larger than 1/φ, which well explicates associated experiments [16,17,18,19].

Not long ago, taking advantage of the highly stressed silicon nitride material, some exceptions breaking the 1/φ limiting rule to a considerable degree have been reported. The membrane [14] resonators applied with a patterned phononic crystal have acquired, extraordinarily, Q > 10^8^ at 1 MHz. Simultaneously, by localizing a central-defect mode away from its supports with soft-clamping phononic crystal, along with tapered geometric strain engineering to realize stress localization, the Q of string [20] resonator reached up to 800 million with nanogram effective masses [21,22,23,24]. Thus far, it is explicated [16,17,25] that the pre-stress seems to “dilute” the intrinsic energy dissipation, bringing about marvelous coherence, which makes several quantum effects possible to realize even at room temperatures with nanomechanical [26,27,28,29]. Otherwise, according to the dissipation theorem, the dissipation dilution effect could be better if decreasing device dimension, implying that the smaller the resonators, the higher the quality factors. This unusual scaling well explains why the quality factor of SiN nanomechanical resonator is extraordinarily high [21,30,31,32].

However, most articles concentrated on the SiN material. Compared to SiN, graphene has a larger Young’s modulus of 1 TPa, about a forth more than that of SiN, which means that graphene could hold much more stress than SiN under the same condition. Meanwhile, above all else, single-layer graphene’s thickness is 0.335 nm, which is one hundredth of silicon nitride’ typical thickness about 30 nm, implying that the dissipation dilution effect of graphene could be much better than that of silicon nitride.

## 2. Methodology

### 2.1. Analytical Model

According to dissipation dilution theory, the effect of phononic crystal suppressing radiation loss can be well captured by a Zener model. In this model, we could obtain the total loss per cycle, also treated as bending energy [16,33,34].

(1) $$\Delta U=\frac{\pi E_{2}h^{3}}{12(1-\nu^{2})}\int \int (\frac{\partial^{2}W}{\partial x^{2}}+\frac{\partial^{2}W}{\partial y^{2}}{)}^{2}dxdy$$

in which *W*(*x*, *y*) is the out-of-plane displacement, *h* is plate thickness and *ν* is Poisson’s ratio. During the formula derivation, a complex-valued Young’s modulus is introduced, resulting in a phase lag between the strain and stress: $\tilde{E}=E_{1}+iE_{2}$. For each oscillating cycle, the mode’s total energy, also regarded as elongation energy, can be expressed as [16].

(2) $$U_{mn}=\frac{\rho h\omega_{mn}^{2}}{2}{\int \int \left(W(x,y)\right)}^{2}dxdy=2\rho \pi^{2}f_{mn}^{2}{\int \left(W(x,y)\right)}^{2}dV$$

in which *ω_mn_* is the resonant angular frequency, and *f_mn_* is the resonant frequency. Then for each mode, we could obtain its quality factor [35,36].

(3) $$Q_{mn}=\frac{2\pi U_{mn}}{\Delta U}=A_{mn}\times Q_{\mathrm{int}}$$

(4) $$A_{mn}=\lambda_{mn}^{4}\times \frac{{\int \int \left(W(x,y)\right)}^{2}dxdy}{\int \int {\left(\frac{\partial^{2}W}{\partial x^{2}}+\frac{\partial^{2}W}{\partial y^{2}}\right)}^{2}dxdy}\;\lambda_{mn}^{2}=\omega_{mn}\sqrt{\rho h/D}$$

in which *A_mn_* is the quality factor amplification coefficient, *Q*_int_ is the intrinsic material quality factor $Q_{\mathrm{int}}=E_{1}/E_{2}$, and *D* is the flexural rigidity: $D=E_{1}h^{3}/12\left(1-\nu^{2}\right)$.

For an isotropic thin plate with uniform boundary tension and under sinusoidal oscillation in the transverse direction, the dimensionless differential equation for a mode *W*(*x*, *y*) is:

(5) $$\nabla_{}^{4}W(x,y)-\frac{T}{D}\nabla_{}^{2}W(x,y)-\lambda^{4}W(x,y)=0$$

in which *T* is tension, *σ* is stress:

(6) T=σ×h

With the condition that all boundary sides of the thin plate are clamped:

(7) $$W(x,y){∥}_{\mathrm{all}\mathrm{edges}}=\frac{\partial W(x,y)}{\partial n}{∥}_{\mathrm{all}\mathrm{edges}}=0$$

Here *n* is the axis perpendicular to the edge. In this differential equation for a specific mode, the amplification factor *A_mn_* in Equation (4) could be separated into a distributed part and a clamping part. The distributed part defines the area away from the clamping boundry. In order to find the distributed energy, we neglect the first bending term in Equation (5), which only weakly perturbs the solution in the region away from the clamping points. Take the simplified Equation (5) into Equation (4), then the distributed part can be derived as:

(8) $$\begin{array}{ll} & -\frac{T}{D}\nabla_{}^{2}W(x,y)-\lambda^{4}W(x,y)=0\\ A_{mn}^{distributed} & =\lambda_{mn}^{4}\frac{{\int \int \left(W(x,y)\right)}^{2}dxdy}{\int \int {\left(\nabla_{}^{2}W(x,y)\right)}^{2}dxdy}=\frac{1}{{\left(\lambda_{mn}^{2}D/T\right)}^{2}} \end{array}$$

Meanwhile, for the clamping part, *W*(*x*, *y*) is close to 0, so that the last term on the left of Equation (5) can be neglected. Taking the simplified Equation (5) into Equation (4), then the clamping part can be derived as:

(9) $$\begin{array}{c} \nabla_{}^{4}W(x,y)-\frac{T_{clamp}}{D}\nabla_{}^{2}W(x,y)=0\\ A_{mn}^{clamp}=\lambda_{mn}^{4}\frac{\iint_{cla}{\left(W(x,y)\right)}^{2}dxdy}{\int \int {\left(T_{clamp}/D\right)}^{2}dxdy}=\frac{\lambda_{mn}^{4}}{S{\left(T_{clamp}/D\right)}^{2}}\iint_{cla}{\left(W(x,y)\right)}^{2}dxdy \end{array}$$

in which *S* is the area of the thin plate, while the abbreviation of *cla* delegates merely integrating the clamping area. The overall quality factor amplification factor can be calculated as:

(10) $$A_{mn}={\left[{\left(A_{mn}^{distributed}\right)}^{-1}+{\left(A_{mn}^{clamping}\right)}^{-1}\right]}^{-1}={\left[{\left(\lambda_{mn}^{2}D/T\right)}^{2}+\frac{S{\left(T_{clamp}/D\right)}^{2}}{\lambda_{mn}^{4}\iint_{cla}{\left(W(x,y)\right)}^{2}dxdy}\right]}^{-1}$$

Using a phononic crystal structure could compress the clamping dissipation part to a very small value on this condition: $A_{mn}\approx A_{mn}^{distributed}$. For a square isotropic thin plate with a side length of *L* and uniform stress of *σ*, the oscillating frequency can be calculated as [17]:

(11) $$\omega_{mn}^{2}=\omega_{m}^{2}+\omega_{n}^{2}\approx {\left(\frac{m\pi }{L}\sqrt{\frac{\sigma }{\rho }}\right)}^{2}+{\left(\frac{n\pi }{L}\sqrt{\frac{\sigma }{\rho }}\right)}^{2}=\frac{\sigma \pi^{2}}{\rho L^{2}}\left(m^{2}+n^{2}\right)$$

Then the quality factor amplification coefficient of a square isotropic thin plate with a phononic crystal structure can be derived as:

(12) $$A_{mn}\approx A_{mn}^{distribution}=\frac{T^{2}}{\omega_{mn}^{2}\rho hD}\approx \frac{T^{2}}{\rho hD}{\left[\frac{\sigma \pi^{2}}{\rho L^{2}}\left(m^{2}+n^{2}\right)\right]}^{-1}=\frac{12\sigma L^{2}\left(1-v^{2}\right)}{E_{1}h^{2}\pi^{2}\left(m^{2}+n^{2}\right)}$$

From Equation (12), the amplification factor is proportional to stress *σ* and the square of the side length, *L*^2^, while inversely proportional to the square of thickness *h*^2^ and mode number (*m*^2^ + *n*^2^).

According to Equation (12), we could make a comparison between SiN and graphene. The following material parameters were considered for the SiN membrane: density *ρ* = 3200 kg/m^3^, thickness *h* = 30 nm, Young’s modulus *E*_1_ = 270 GPa and Poisson ratio *ν* = 0.27 [22] and for the graphene film: density *ρ* = 2250 kg/m^3^, thickness *h* = 30 nm, Young’s modulus *E*_1_ = 1 TPa and Poisson ratio *ν* = 0.165 [37]. Supposing that both materials are under the same stress *σ*, size *L* and mode number (*m*^2^ + *n*^2^), the quality factor amplification coefficient of graphene would be 2833 times that of SiN, not to mention that graphene’s Young’s modulus is about forth more than that of SiN. The breaking strength *σ*_break_ of graphene is much higher than that of SiN, meaning that graphene could hold much more stress leading to a larger amplification coefficient. However, according to Equation (3), the quality factor *Q*_mn_ is not only dependent on the amplification coefficient *A*_mn_ but also related to the intrinsic material quality factor *Q*_int_. The typical *Q*_int_ value of SiN is about 2000 with a 30 nm thickness at room temperature, approximately 10 times that of graphene. To sum up, the overall quality factor *Q*_mn_ of graphene is about 283 times that of SiN.

### 2.2. Computer Simulations

**Figure 1:**
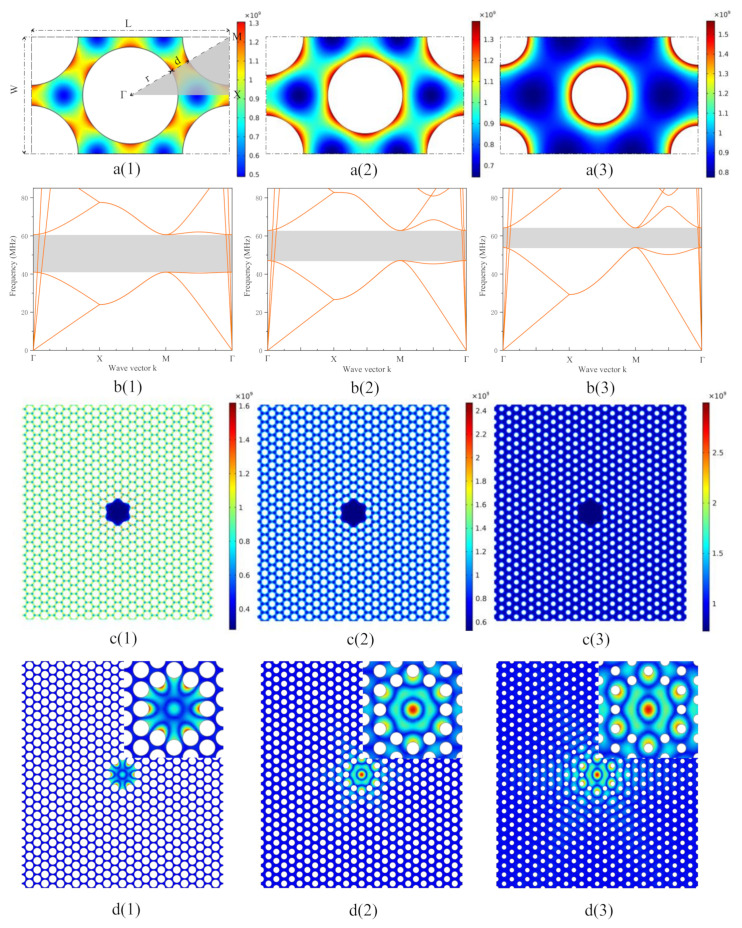
**a(1)**–**a(3)** Three periodic primitive cells with different interval gaps *d* as: 1 μm, 2 μm and 3 μm, along with the simulation results of the stress redistribution. The grey triangular area interprets the collective first Brillouin zone. **b(1)**–**b(3**) Quasi-bandgaps are opened, marked with grey area, corresponding to **a(1)**–**a(3)** respectively. **c(1)**–**c(3)** Realistic devices of finite size made up from three different triangular lattice unit cells accompanied by stress redistribution, corresponding to **a(1)**–**a(3)** respectively. **d(1)**–**d(3)** Localized vibrational shapes of the first defect modes originating from three structures, the insets amplify the central defect areas, corresponding to **a(1)**–**a(3)** respectively.

Through finite element simulation, we further confirm this conclusion. We used COMSOL Multiphysics to simulate the phononic crystal patterned membrane resonators. The simulation parameters are the same as the theoretical calculation part above with the initial stress *σ* of 1 GPa for both materials. To minimize numerical errors, the geometry was densely meshed. The simulations were typically carried out in two steps. First, we performed a stationary study to calculate the stress redistribution caused by perforation, assuming a homogeneous initial in-plane stress *σ*_xx_ = *σ*_yy_ whether in an infinite array cell with graphene material, shown as Figure 1a(1), or in the actual device consisting of 13 × 25 periodic triangular lattice primitive cells with a central defect area, shown as Figure 1c(1). The detail cell sizes in the Figure 1a(1) are as follows: lattice constant *L* = 10 μm, interval gap *d* = 1 μm and $W=L/\sqrt{3}$, $r=\left(W-d\right)/2$. The redistributed stress was subsequently used in an eigenfrequency analysis, in which we either calculated the eigenmodes of an infinite array for different wavevectors *k* in the first Brillouin zone with floquet periodic boundary conditions, shown as Figure 1b(1), or simply simulated the eigenmodes of the actual devices with fixed boundary conditions, shown as Figure 1d(1).

**Figure 2:**
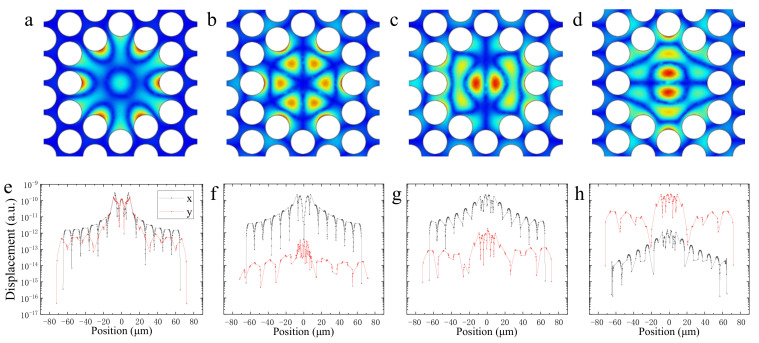
(**a**–**d**), Mode shapes of the four localized defect modes; (**e**–**h**) corresponding projections of the absolute value of displacement fields along the x- (black) and y-directions (red).

The mechanical quality factors were extracted by calculating the curvature of a given localized mode, which is obtained from an eigenfrequency simulation, as described above in Equation (3). In the simulation results, we finally obtain four central defect modes with substantial out-of-plane displacements within the bandgap. Mode shapes of the four localized defect modes are showed as Figure 2a–d, merely shown the central defect areas wrapped by several cells. Their corresponding projections of displacement fields along x-direction (black) and y-direction (red) crossing the structure’s central point are depicted in Figure 2e–f, respectively, in the form of absolute value. We can find that the vibration amplitude in the central defect region is several orders of magnitude higher than that around the clamping edge area, implying that the soft-clamping phononic crystal has well accomplished localizing the central-defect mode away from its supports. Among the four defect modes, the quality factor of the first one is the highest. In following simulation, we take the first central defect mode as the default one. For the SiN material, the simulation result is similar to that of graphene material but different in numerical values. For the default resonant mode defined above, the amplification coefficients A of graphene and SiN are about 3.02 × 10^5^ and 151, respectively, with the division result of about 2002 times, while the quality factors are about 6.04 × 10^7^ and 3.02 × 10^5^, respectively, with the division result of about 200 times, approximating but lower than the theoretical calculation results that the amplification coefficient A of graphene is 2833 times that of SiN, while the quality factor is 283 times. We attribute the approximate 20% discrepancy to the clamped radiation losses in finite simulation, against which these modes are not protected in theoretical calculation. 

## 3. Results and Discussion

To maximize the quality factor, we further optimize the phononic crystal geometric structure. First, we keep the length *L* and width *W* of the primitive cell unchanged and vary the value of interval gap between two adjacent round holes *d* as the following: 1 μm, 2 μm and 3 μm. We use ratio between the interval gap and lattice constant *d*/*L* to define the duty cycle of phononic crystal symbolizing the triangular lattice cell shape, as depicted in Figure 1; the (1)–(3) represent the three *d* values, respectively. Figure 1a(1)–a(3) shows the structure of three different periodic primitive cells, with the simulation results of the stress redistribution in a unit cell of the triangular lattice. The grey triangular area in Figure 1a(1) interprets the collective first Brillouin zone for all three cell structures. The initial stress *σ*_0_ applied to three unit cells is 1 GPa, and after stress redistribution the minimum and maximum value reach 0.5 and 1.6 GPa, respectively.

A full bandgap is not expected here because of the extreme ratio *h*/a ≤ 10^−6^, as shown in Figure 1b(1)–b(3). A quasi-bandgap can nonetheless be opened, whereby only in-plane modes with a high phase velocity persist in the gap. Under a high tensile stress, the triangular lattice achieves a relatively large bandgap about 40% of the central frequency 5 × 10^7^ Hz marked with a grey area as shown in Figure 1b(1), with a hole radius of *r* ≈ 0.24 *L*. With the increasing *d*, the *r* decreases to 0.19 *L* and 0.14 *L*, while the bandgap decreases to about 29% and 17% of the central frequency of 5.5 × 10^7^ and 5.9 × 10^7^ Hz as shown in Figure 1b(2),b(3), respectively. At the same time, the design allows the phononic crystal structure to be realized relatively easily via Photolithography, Focused Ion Beam Etching or other relevant micro-nano techniques, given that the tether width is still above 1 μm even for the smallest structure. Apparently, the phonon dispersion is modified significantly by the in-plane stress, which relaxes to an anisotropic and inhomogeneous equilibrium distribution that must be simulated beforehand.

To study a realistic device of finite size and to implement a defect into the phononic pattern, we conducted a second independent simulation (“finite model”). In this model, we considered a finite number of unit cells of the triangular lattice (same as the *W*, *L*, *d* and *σ*_0_ as before) and employ fixed boundary conditions along the phononic crystal’s perimeter depicted in Figure 1c(1)–c(3). First, we simulated the relaxation process of an anisotropic and inhomogeneous stress equilibrium distribution, leading to the stress redistribution shown as Figure 1c(1)–c(3). Next, an eigenfrequency study was implemented. Within the bandgap, the localized vibrational shapes of the first defect mode are depicted in Figure 1d(1)–d(3) with substantial out-of-plane displacements. Inset is a magnification of the defect area, wrapped by several primitive cells.

With the full simulated displacements at hand, we were in a position to evaluate the bending energy in Equation (1) and the total stored energy in Equation (2). According to Equation (4), we could calculate the quality factor amplification coefficients of all three localized defect modes originating from different *d* values in Figure 1d(1)–d(3). For *d* = 1 μm, the quality factor amplification coefficient was about 3.02 × 10^5^ resonating at 4.96 × 10^7^ Hz, while for *d* = 2 μm and *d* = 3 μm, the amplification coefficients were about 2.25 × 10^5^ and 2.03 × 10^5^ resonating at 5.76 × 10^7^ Hz and 6.37 × 10^7^ Hz, respectively. Then a preliminary conclusion can be drawn that the smaller the *d*, the bigger the duty cycle and the larger the quality factor amplification coefficient.

**Figure 3:**
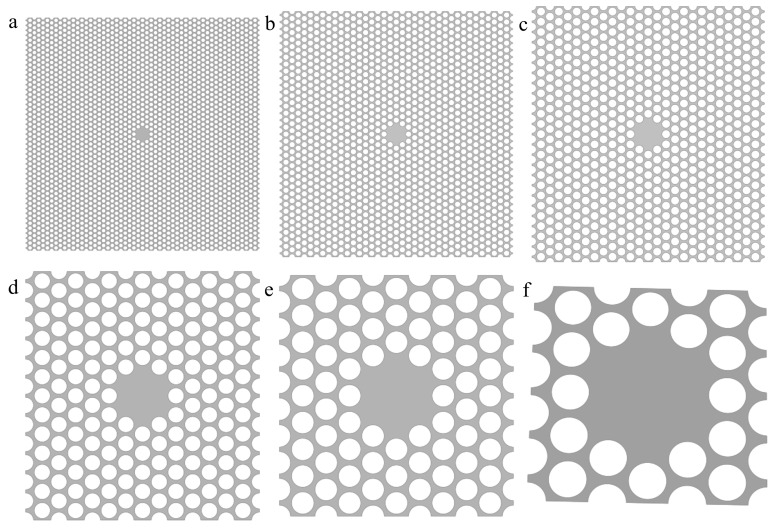
(**a–f**) The actual devices are consisting of 27 × 47, 19 × 35, 13 × 25, 7 × 13, 5 × 9 and 3 × 5 different-lattice-constants *L* but same-duty-cycle *d*/*L* cells with central defect areas, respectively, meanwhile fixing the overall width unchanged.

In following optimization procedure, we altered the *L* value to pursue the optimal structure, meanwhile fixing the ratio between *d* and *L* value as 1/10, also treating as the duty cycle defined above. For better comparison, the overall width remains unchanged as 130 μm. From Figure 3a–f, the actual devices are consisting of 27 × 47, 19 × 35, 13 × 25, 7 × 13, 5 × 9 and 3 × 5 different-lattice-constants but same-duty-cycle cells with central defect areas. In order to maintain the structural symmetry in the horizontal and vertical directions, meanwhile, to keep the overall width consistent, the same length has to be sacrificed among all six structures.

**Figure 4:**
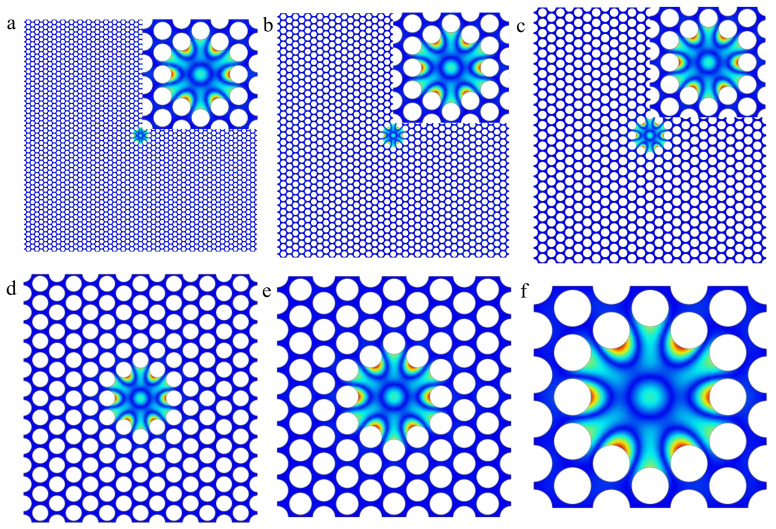
(**a**–**f**), The first defect mode shapes correspond to Figure 3a–f, respectively. Their out-of-plane displacements are approximately in the same vibration morphology.

Through a stationary study to calculate the stress redistribution and a subsequent eigenfrequency analysis to calculate the eigenmodes of both an infinite array for different wavevectors *k* and the actual devices with fixed boundary conditions, the corresponding first defect mode shapes are depicted in Figure 4a–f, respectively, in which we find that their out-of-plane displacements are approximately in the same vibration morphology. The insets in Figure 4a–c represent the magnification of the defect areas, wrapped by several primitive cells.

**Figure 5:**
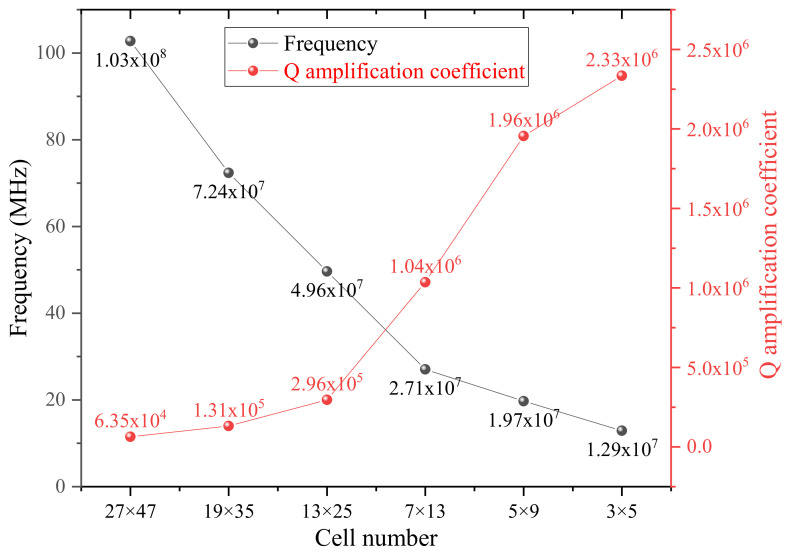
The quality factor amplification coefficients (red) of all six structures along with the resonant frequencies (black). The Q amplification coefficient of the structure making up from the 3 × 5 cells, which is the least number to compose a phononic crystal with a central defect area, is the highest.

According to Equation (4), we then calculate the quality factor amplification coefficients of all six structures with the simulated displacements. The results (red) are illustrated in Figure 5, along with the resonant frequencies (black). Through comparison, to our surprise, the Q amplification coefficient of the structure making up from the 3 × 5 cells, which is the least number to compose a phononic crystal with a central defect area, is the highest, approximately 36 times that of the structure consisting of the 27 × 47 cells, the maximum quantity. It can be interpreted that the central defect area becomes larger as the component cell number decreases. Based on Equation (12), the amplification factor is proportional to the square of side length *L*^2^, which can also be regarded as the area of the actual structure. Meanwhile, the vibration amplitude, as well as the normalized curvature $\left|\left(\partial_{x}^{2}+\partial_{y}^{2}\right)W\left(x,y\right)\right|U_{mn}^{-1/2}$ tightly connected with the amplification coefficient, in the central defect region is several orders of magnitude higher than that surrounding the soft-clamping phononic crystal parts. So, the amplification coefficient of the whole structure can be considered approximately equal to that of the central defect area, which well explains why the minimal cell-number structure could possess the maximum quality factor. Preliminary conclusions can be drawn that under the same duty cycle and overall width of the actual devices, the larger the lattice constant *L*, the less the cell number making up the structure, the larger the Q amplification coefficient. Moreover, for the device consisting of 3 × 5 cells, the first defect mode happens to locate in the 31st mode counted from the fundamental mode, which can be regarded as a very low mode order. In contrast, the first defect mode orders of the remaining five structures are ranked in the hundreds. In addition, the more cells, the higher the modal order of the first defect mode, which also can be perceived from the increasing resonance frequency from right to left in Figure 5.

**Figure 6:**
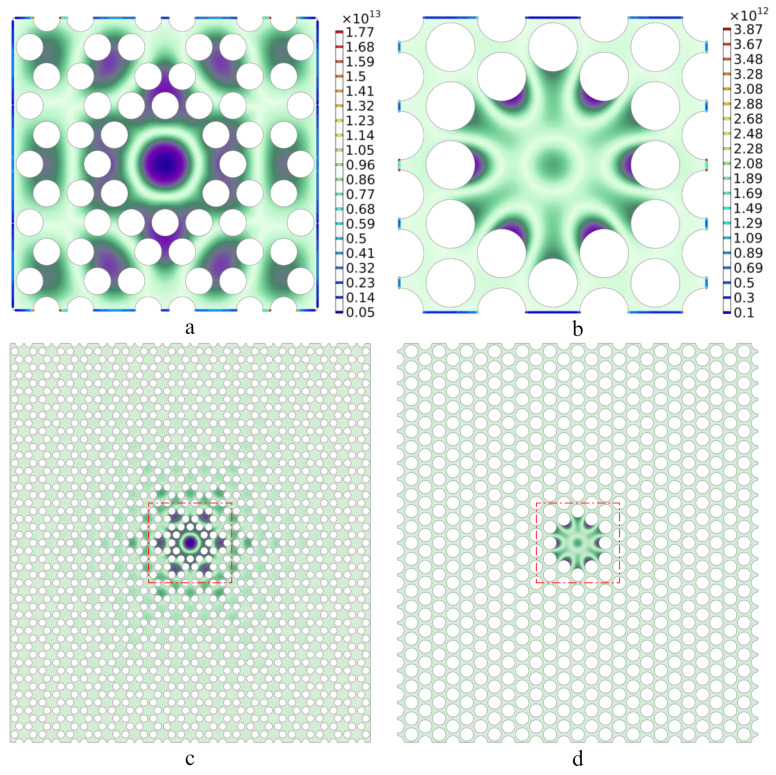
(**a**,**b**) The AuroraBorealis color (green and purple) depicts the first defect mode shape of the minimal cell-number structure with central defect in the hexagonal lattice and triangular lattice. The Rainbow color (blue and red) describes the normalized curvature in the fixed boundary area, with the legend on the right panel; (**c**,**d**) the first defect mode shapes of the 13 × 25-cell structure with central defect in the hexagonal lattice and triangular lattice. The region enclosed by the red dotted line frame is in the same geometry and size with (**a**,**b**), respectively.

However, the minimal cell-number structure with a central defect in a hexagonal lattice [21] could not achieve the brilliant dissipation dilution effect as well as the triangular lattice. As shown in Figure 6a,b, the AuroraBorealis color (green and purple) depicts the first defect mode shape of the minimal cell-number structure with central defects in the hexagonal lattice and triangular lattice, respectively. Both structures consist of the 3 × 5 primitive cells, same in the size but different in the lattice morphology. The Rainbow color (blue and red) describes the normalized curvature in the fixed boundary area, since the normalized curvature of the interior part far away from the border is relatively small, so we merely consider the normalized curvature of the clamping region. As can be seen from the legend on the right panel, the normalized curvature of the hexagonal lattice is an order of magnitude higher than that of the triangular lattice, implying that the amplification coefficient of the former is much smaller than that of the latter, based on the inverse square relationship between the amplification coefficient and the normalized curvature: $A_{mn}^{-1/2}\infty \left|\left(\partial_{x}^{2}+\partial_{y}^{2}\right)W\left(x,y\right)\right|U_{mn}^{-1/2}$. Through precise calculation, the amplification coefficient of the hexagonal lattice is about 5.17 × 10^4^, while the result of the triangular lattice can be obtained from Figure 5 as 2.33 × 10^6^, approximately, with the division value about 45 times. Comparing to more cell-number structure further confirms this conclusion. Figure 6c,d shows the first defect mode shapes of the 13 × 25-cell structure with central defects in the hexagonal lattice and triangular lattice, respectively. The part enclosed by the red dotted line frame in Figure 6d is equal to Figure 6b, where their out-of-plane displacements are approximately in the same vibration morphology as discussed above, indicating that the minimal cell-number structure could achieve the excellent dissipation dilution effect as well as the one making up from more primitive cells. However, the red dotted line frame in Figure 6c cannot nicely wrap all the vibrating defect regions; outside the red dotted line frame, there are still non-negligible parts with out-of-plane vibrations. Meanwhile, although the region enclosed by the red dotted line frame is in the same geometry and size with Figure 6a, it is slightly different in the modal shape, especially losing the hexagonal symmetry in the horizonal direction. This phenomenon further corroborates that the dissipation dilution effect of minimal cell-number structure with central defects in the triangular lattice is much better than that in the hexagonal lattice.

**Figure 7:**
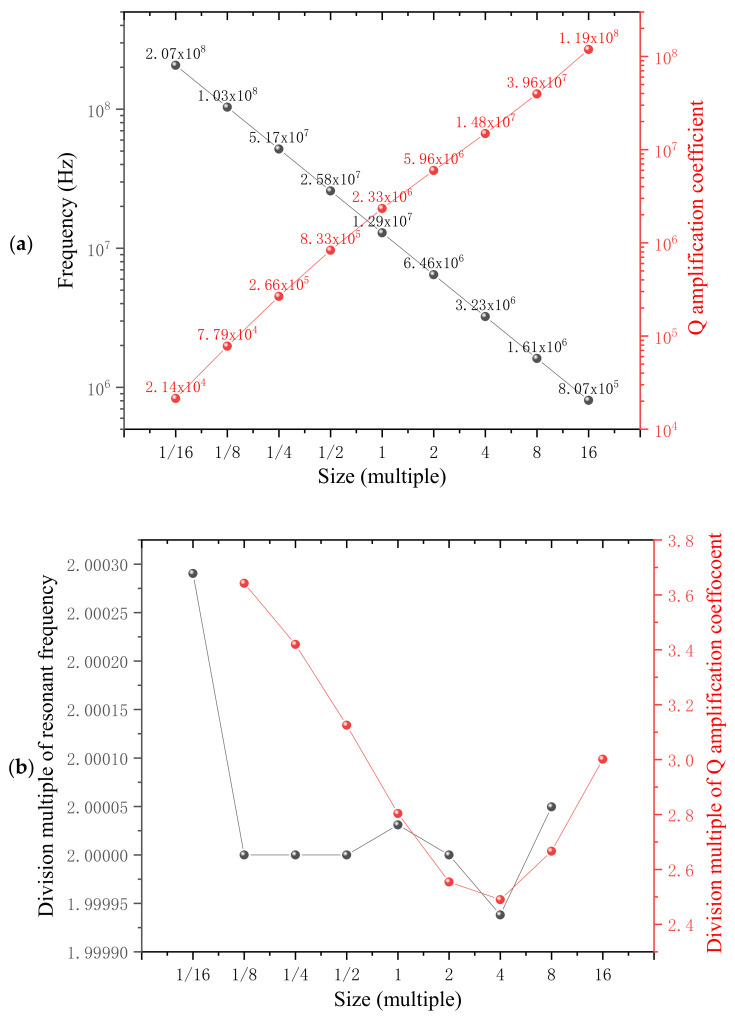
(**a**), The relationship between the overall size and Q amplification coefficient (red) along with the resonant frequency (black) in the form of logarithmic coordinates. (**b**) Division multiples between adjacent two resonance frequencies as well as Q amplification coefficients.

Then, we took the 3 × 5-cell structure as the next research object to study how the stress and overall size influence the Q amplification coefficient. The initial 3 × 5-cell structure was about 130 μm × 125 μm in size; we expanded the overall size by 2, 4, 8 and 16 times and reduced the overall size to half, a quarter, one 8th and one 16th. Through similar simulation processes, the relationship between the overall size and Q amplification coefficient (red) was obtained as Figure 7a along with the resonant frequency (black) in the form of logarithmic coordinates. For better comparison, Figure 7b describes the division multiple between two adjacent resonance frequencies as well as Q amplification coefficients. The division multiples of resonance frequency are around two, consistent with the theoretical calculation based on Equation (11). However, the division multiples of Q amplification coefficients are scattered between 2.4 and 3.8. According to Equation (12), the Q amplification coefficient and the *L* symbolizing the overall size are in a square relationship: $A_{mn}\infty L^{2}$, meaning that the finite simulation value is deviated from the theoretical calculation result of four times.

**Figure 8:**
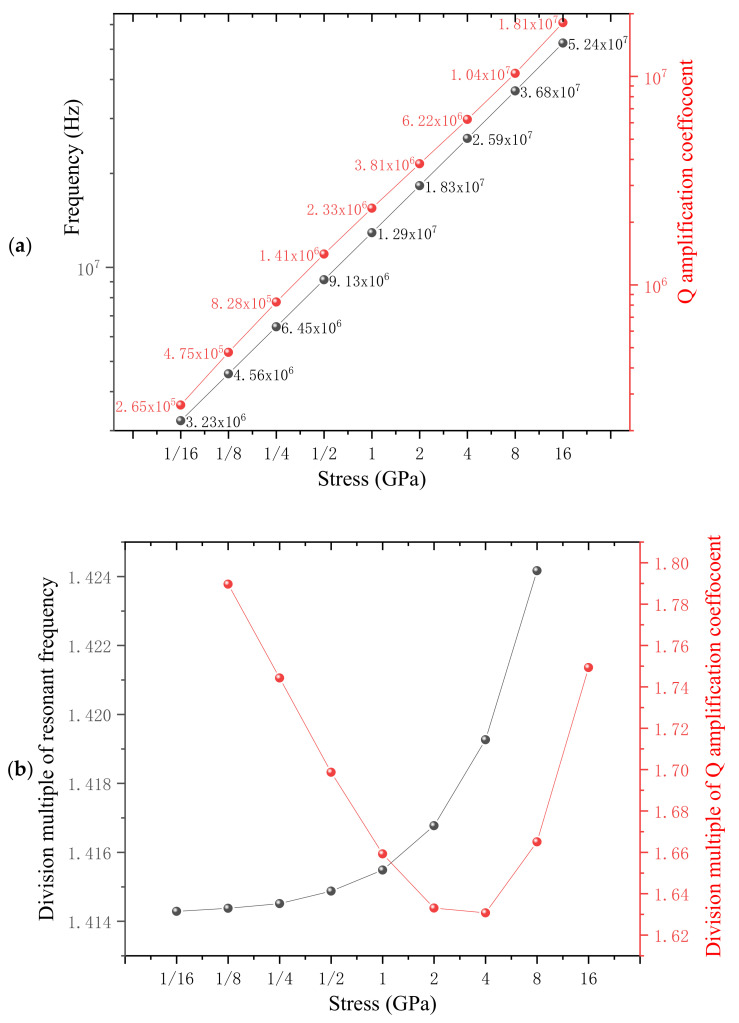
(**a**), The relationship between the stress and Q amplification coefficient along with the resonant frequency both in the form of logarithmic coordinates. (**b**) Division multiples between adjacent two resonance frequencies as well as Q amplification coefficients.

The initial homogeneous in-plane stress is 1 GPa, to study the relationship between stress and Q amplification coefficient, it is increased in double equal proportion between one sixteenth and sixteen GPa. The simulation results are depicted in the form of logarithmic coordinates in Figure 8a, in which resonant frequency and Q amplification coefficient increase in pace with the enlargement of stress. For more convenient contrast, the division multiples between adjacent two ordinate values from Figure 8a are displayed in Figure 8b. The division multiples of resonance frequencies fluctuate slightly near 1.414 ($\sqrt{2}$), in consistent with the theoretical calculation result according to Equation (11). However, all division multiples of Q amplification coefficients are slightly lower than the theoretical calculation result of 2 times based on Equation (12), in which the Q amplification coefficient is linear to the stress: $A_{mn}\;\infty \;\sigma$.

**Figure 9:**
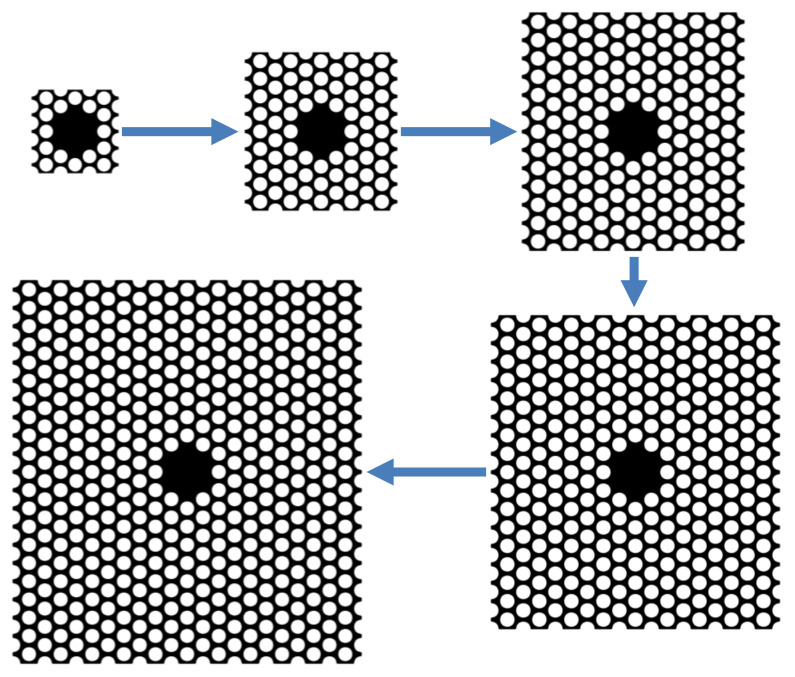
Start from the primitive 3 × 5-cell structure with central defect, constantly adding cells to the periphery, changing the structure composition into 5 × 9, 7 × 13, 9 × 17, 11 × 21 cells along the direction of the blue arrow.

**Figure 10:**
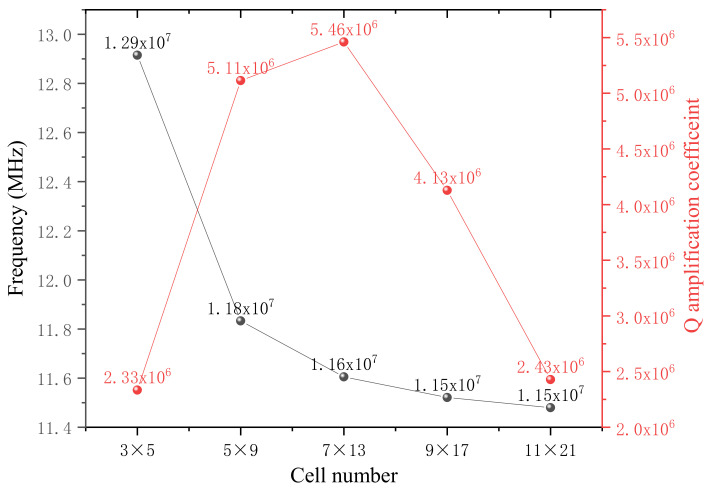
The correlation between cell number and Q amplification coefficient as well as the resonant frequency. The largest Q amplification coefficient does not belong to the largest structure, instead originating from the moderate one consisting of the 7 × 13 cells.

At last, for the sake of testing how many cells could achieve relatively better dissipation dilution effect deriving from the phononic crystal soft clamping, we start from the primitive 3 × 5-cell structure with central defect, 130 μm × 125 μm in size with 1 GPa homogeneous in-plane stress. While keeping the central defect region unchanged, primitive cells are constantly added to the periphery as showed in Figure 9, along the direction of the blue arrow, changing the structure composition into 5 × 9, 7 × 13, 9 × 17 and 11 × 21 cells. The biggest structure size reaches about 477 μm × 525 μm. Through simulation, surprisingly, the largest Q amplification coefficient does not belong to the largest structure, instead originating from the moderate one consisting of 7 × 13 cells, which is about twice the minimum value as depicted in Figure 10. Multiplied by graphene’s intrinsic quality factor 200 at ambient temperature, based on Equation (3), eventually the actual quality factor could reach as high as 10^9^ magnitude, which could be regarded as a remarkably large value for an approximate 0.25 mm^2^ resonant membrane. At the same time, we can also find that the resonant frequency gradually tends to be a stable value of about 1.15 × 10^7^ Hz, indicating that the dissipation dilution effect conceiving from the phononic crystal soft clamping steadily reaches a state of saturation.

## 4. Summary and Conclusions

In summation, according to the Zener model, single-layer graphene’s quality factor is two orders of magnitude larger than that of silicon nitride, either in the manner of theoretical calculation or finite simulation, due to the fact that the thickness of graphene is merely one percent of that of SiN. Starting from the triangular lattice phononic crystal, we further optimized the structure in terms of duty cycle and cell size to achieve higher quality factors. Through finite simulation analysis, a preliminary conclusion was drawn that the smaller the *d*, the bigger the duty cycle, the lower the defect-mode resonant frequency, the wider the bandgap and the larger the quality factor amplification coefficient. Next, we took the cell size *L* into consideration. Through comparison, unexpectedly, the Q amplification coefficient of the structure making up from the 3 × 5 cells with the biggest cell size, which is the least number to compose a phononic crystal with a central defect area, was the highest. We attribute it to the reason that the central defect region becomes larger with the decreasing component cell number. Moreover, the first defect mode happened in the 31st mode, which can be regarded as a very low modal order for phononic crystal. Meanwhile, we contrasted it with the hexagonal lattice. Simulation results show that the minimal cell-number structure with a central defect in the hexagonal lattice could not achieve the brilliant dissipation dilution effect as well as that in the triangular lattice.

Based on the 3 × 5-cells, we studied how the overall size and stress affect the quality factor. The simulation results coincide with the theoretical calculation but with a lower deviation, in which the Q amplification coefficient was squared to the overall size while linear to the stress. Ultimately, to test how many cells could achieve relatively better dissipation dilution effect, we constantly added cells to the periphery of the primitive 3 × 5-cell structure. Through precise finite simulation, extraordinarily, the largest Q amplification coefficient did not happen in the largest structure, instead arising from the moderate one consisting of the 7 × 13 cells. Simultaneously, we noticed that the resonant frequency progressively converges to a stable value, implying that the dissipation dilution effect gradually reaches a saturation state. In future work, we will make an endeavor to study on relevant MEMS processes to manufacture the perforated graphene membrane resonator with triangular lattice phononic crystal pattern. Correspondingly, associated experiments would be carried out to verify the validity of the structural optimization process, which will be a big challenge.
